# The independent and combined effects of single-child status and ideal lifestyle on clustered cardio-metabolic risk factors among Chinese children and adolescents

**DOI:** 10.3389/fnut.2022.987334

**Published:** 2022-08-29

**Authors:** Jiajia Dang, Ning Ma, Yunfei Liu, Panliang Zhong, Di Shi, Shan Cai, Yanhui Dong, Zhiyong Zou, Yinghua Ma, Yi Song, Jun Ma

**Affiliations:** ^1^Institute of Child and Adolescent Health, School of Public Health, Peking University, Beijing, China; ^2^National Health Commission Key Laboratory of Reproductive Health, Peking University, Beijing, China

**Keywords:** single-child, ideal lifestyle, clustered CMRFs, children, adolescents

## Abstract

**Background:**

Cardio-metabolic risk factors (CMRFs) represent the accumulation of metabolic abnormalities, significantly increasing the likelihood of cardiovascular diseases. Although studies assessed the independent association of single-child status and lifestyle risk factors with components of CMRFs or clustered CMRFs, little has been known about the combined effect of single-child status and lifestyles on clustered CMRFs as well as sex differences.

**Materials and methods:**

Data was collected from a cross-sectional survey conducted in September 2013 in China. A total of 13,859 children and adolescents aged 7–18 years with blood samples were included. Anthropometric measurements and serum biochemical indexes were collected to assess clustered CMRFs, while questionnaires were used to obtain single-child status, lifestyle information, and characteristics of children and their parents. Mixed effect logistic regression was applied to analyze the independent and the combined effects of single-child status and ideal lifestyle category on clustered CMRFs.

**Results:**

The prevalence of clustered CMRFs was 3.4%, with a higher prevalence in boys (4.0%) than girls (2.7%). Children and adolescents with clustered CMRFs had a higher proportion of single children (76.6 vs. 69.7%) and unfavorable lifestyles (62.1 vs. 29.2%) compared with their peers with non-clustered CMRFs. Both single children (OR = 1.67, 95% CI: 1.32–2.11) and unfavorable lifestyles (OR = 9.03, 95% CI: 6.26–13.02) were associated with an increased risk of clustered CMRFs. The risk of clustered CMRFs increased significantly (OR = 12.79, 95% CI: 6.67–24.52) when single children and an unfavorable lifestyle were combined, which was almost neutralized (OR = 1.33, 95% CI: 0.63–2.82) when single children adhered to a favorable lifestyle. However, no sex differences were observed in this study.

**Conclusion:**

Single children with unfavorable lifestyles were associated with an obvious risk of clustered CMRFs, which might be partially offset by expanding family size (the number of siblings) or establishing a favorable lifestyle. A birth-friendly social environment as well as a family environment with a favorable lifestyle are encouraged in China.

## Introduction

Cardiovascular diseases (CVDs) are the leading cause of death globally, accounting for 32% of all deaths (1), while in China this proportion has exceeded 40%, accompanied by an age-standardized mortality rate for males being 1.5 times higher than females (2). Cardio-metabolic risk factors (CMRFs) represent the accumulation of metabolic abnormalities, including hypertension, dyslipidemia, hyperglycemia, and abdominal obesity, while clustered CMRFs are defined as meeting at least three of these four abnormalities, significantly increasing the likelihood of CVDs (3). Although CMRFs are more common in adults, a substantial body of research indicated that pre-CMRFs and behavioral patterns were formed in childhood and adolescence (4–6), and that adverse levels of clustered CMRFs often coexisted in the same individual (7). Therefore, identifying modifiable factors for clustered CMRFs in children and adolescents is becoming to be recognized as a useful strategy to reduce the lifetime incidence of CVDs.

In the past few decades, China has undergone a remarkable shift in family structure under the implementation of the one-child policy since 1979, which resulted in the birth of more than 100 million families with single children (8). At the same time, the prevalence of cardiovascular and metabolic diseases skyrocketed (9). Based on this, many studies have noted the impact of different family structures on CMRFs, as well as sex differences. Recent studies provided evidence for the association of single children with components of CMRFs such as dyslipidemia (10), abdominal obesity (11), and hypertension (12) in children and adolescents, however, the findings were inconsistent in the direction of the association and sex differences. In addition, only one Iranian study explored the association between single children and all components of CMRFs, and this positive association was mainly manifested in abdominal obesity (13). These evidences suggested that single-child status might play an important role in CMRFs, with varied effects in different sexes. However, so far, studies have focused on the effects of single-child status on the single/multiple components of CMRFs rather than clustered CMRFs.

Furtherly, previous studies found that alcohol intake (14), sedentary behavior (15), insufficient sleep duration (16) and dairy intake (17) all played a significant role in clustered CMRFs. The high prevalence of these behavioral risk factors in childhood and adolescence has been a major concern because they are often maintained to adulthood (18, 19). However, the majority of CVDs can be avoided by addressing behavioral risk factors like tobacco and alcohol consumption, unhealthy diet, obesity, and lack of physical activity (1), which epidemiological studies has validated (20, 21), and so did expect to work for clustered CMRFs.

Although studies have shown that single children are more likely to establish an unfavorable lifestyle (22, 23), little has been known about the interaction of single-child status and lifestyles on clustered CMRFs as well as sex differences. In the real world, it is critical to comprehend the combined impact of disease risk factors, particularly those that coexist or cluster in the same individual, such as CMRFs. In this case, determining the combined impact of its risk factors enables a greater emphasis on controlling modifiable risks and, as a result, better prevention. Therefore, in this study, we hypothesized that there was a combined effect of single children and lifestyles on clustered CMRFs in children and adolescents, and this effect differed by sex. We aimed to investigate the independent and combined effects of two risk factors, single-child status and lifestyles, on clustered CMRFs among children and adolescents aged 7–18 years and the sex differences in these effects using data from a cross-sectional study in China in 2013.

## Materials and methods

### Study design and participants

**FIGURE 1:**
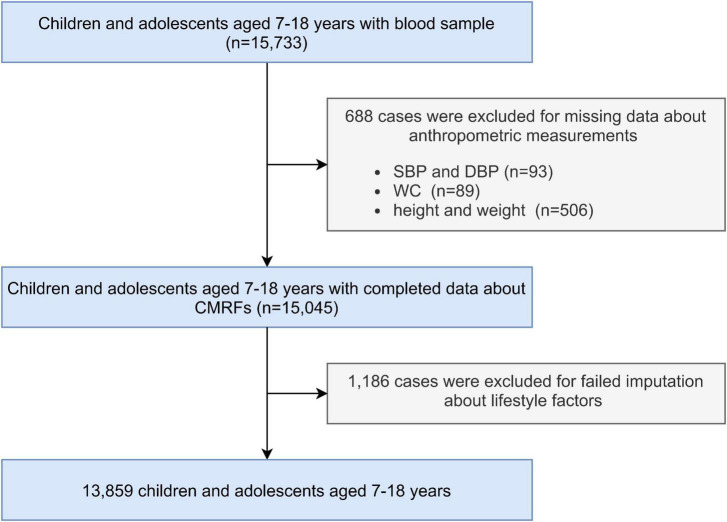
Flow chart of participants’ inclusion. SBP, systolic blood pressure; DBP, diastolic blood pressure; WC, waist circumference; CMRFs, cardio-metabolic risk factors.

Data was collected from a cross-sectional survey conducted in September 2013 with multi-stage cluster random sampling method, involving seven provinces (Liaoning, Ningxia, Tianjin, Shanghai, Hunan, Chongqing, and Guangdong) in China. The specific sampling and implementation methods have been described previously (24). A total of 15,733 children and adolescents aged 7–18 years with blood samples were included. Participants with missing data about anthropometric measurements and failed imputation of missing variables were excluded. The remaining 13,859 children and adolescents were included in the final analysis (Figure 1).

### Data collection and questionnaire survey

#### Anthropometric measurements

All anthropometric measurements were carried out by trained investigators using standardized instruments and procedures, which was described in detail in previous study (24). Briefly, children and adolescents needed to wear light clothing and no shoes when having their body height, weight and waist circumference (WC) measured. Height and WC were measured to the nearest 0.1 cm, and weight was measured to the nearest 0.1 kg. Prior to measuring systolic blood pressure (SBP) and diastolic blood pressure (DBP), participants were required to mediate for 5 mins. All indicators were measured twice, with the mean value being used as the final value.

#### Blood sample collection and detection

After 12 h of fasting, venipuncture was used to draw blood from the veins. Serum was then extracted after centrifugation at 3,000 rpm for 10 min and brought to the testing facility at low temperature (−80°C). A biomedical analysis company with Peking University certification carried out blood biochemical analysis (25). Finally, fasting blood glucose (FBG) and four lipid indicators, including total cholesterol (TC), triglyceride (TG), low-density lipoprotein cholesterol (LDL-C) and high-density lipoprotein cholesterol (HDL-C), were collected.

#### Questionnaire survey

A questionnaire for children and adolescents was used to collect sociodemographic characteristics (age, sex, residence, and school) and lifestyle information such as tobacco and alcohol consumption in the last 30 days, dietary consumption (meat, sugar-sweetened beverage, fruit, and vegetable), physical activity and screen time in the last 7 days, and daily sleep duration. The frequency (day) and amount (servings)/duration (h) were collected for the average daily consumption of each food, as well as daily physical activity and screen time using the following equation: (day × quantity for each day)/7.

A parental questionnaire was used to assess single-child status, which was divided into two categories: single children and non-single children. In addition, a family history of diseases (obesity, hypertension, diabetes, or cerebrovascular disease), parental education, parental tobacco, and alcohol consumption were also gathered.

### Definition and categorization of indicators

#### Clustered cardio-metabolic risk factors

Cardio-metabolic risk factors were defined as hypertension, dyslipidemia, hyperglycemia, and abdominal obesity (26). Hypertension was considered as SBP and/or DBP ≥ the 95th percentile of blood pressure sex- and age-specific group (27). Dyslipidemia was considered as abnormal in any of the following four indicators (28): (I) TC ≥ 5.2 mmol/L; (II) In 0–9 age group, TG ≥ 1.1 mmol/L; In 10–18 age group, TG ≥ 1.5 mmol/L; (III) LDL-C ≥ 3.4 mmol/L; (IV) HDL-C ≤ 1.0 mmol/L. Hyperglycemia was considered as FBG ≥ 5.6 mmol/L (29). Abdominal obesity was considered as WC ≥ the 90th percentile of WC sex- and age-specific group (30). Clustered CMRFs was termed as meeting at least three of the above four items (31).

#### Ideal lifestyle category

Current smoking, current alcohol, dietary consumption, physical activity, sleep duration, screen time and healthy body mass index (BMI) were included to construct an ideal lifestyle score according to the definition of ideal cardiovascular health from the American Heart Association (32) and the Dietary Guidelines for School-age Children in China (33). Current smoking status was classified as smoking in the past 30 days (Yes/No), and current alcohol consumption was similar to smoking. The optimal dietary composition was defined as ≥3 servings of fruit per day (about 100 g per serving), ≥4 servings of vegetables (about 100 g per serving), 2–3 servings of meat products (about 50 g per serving), and <1 serving of sugar-sweetened beverage per week (about 250 mL per serving) according to the Dietary Guidelines for School-age Children in China (33). A healthy diet was described as the consumption of at least three or more foods in prescribed amounts. Adequate physical activity was characterized as 1 h or more of moderate to vigorous activity per day. The cut-off for sleep duration and screen time were 9 and 2 h, respectively. A healthy BMI was classified as lower than the 85th percentile of BMI for sex- and age-specific group (34). Children and adolescents who met each of the seven specified ideal lifestyles were given one point, and the total scores were 7. The ideal lifestyle scores were further divided into three groups as unfavorable lifestyles (0–3 points), intermediate lifestyles (4 points) and favorable lifestyles (5–7 points).

#### The combined subgroups

The single-child status was divided into single children and non-single children. Combining the single-child status and ideal lifestyle category, they can be further divided into six groups, namely the single children and favorable lifestyles, single children and intermediate lifestyles, single children and unfavorable lifestyles, non-single children and favorable lifestyles, non-single children and intermediate lifestyles, and non-single children and unfavorable lifestyles.

#### Covariates

In this study, age, residence, family history of diseases, parental education, parental tobacco, and alcohol consumption were considered as context covariates. A family history of the disease was considered a history of obesity, hypertension, diabetes or cerebrovascular disease in either parent. Parental education level was recorded by the highest educational level of the parents and divided into primary or below, secondary or equivalent and junior college or above. Parental tobacco consumption (Yes/No) was defined as smoking by either parent and parental alcohol consumption (Yes/No) was similar.

### Statistics analysis

Hot-deck Imputation method was used for missing variables (e.g., lifestyle information), and background variables were set as school, grade, urban and rural area, and sex. The quantitative variables were described by median (interquartile spacing), and group differences were tested by Mann-Whitney *U* test according to the normality of distribution. Qualitative variables were described by frequency (percentage) and group differences were tested by Chi-square test. Mixed effect logistic regression was applied to analyze the independent and the combined effects of single-child status and ideal lifestyle category on clustered CMRFs. Only the random effect of schools was adjusted for all crude models. The fixed effect of covariates and the random effect of schools were adjusted in all adjusted models. We also constructed sex interaction terms and conducted stratified analysis to explore sex differences between these association. All analyses were performed in SPSS 26.0 and GraphPad Prism 9. A two-tailed *P* value < 0.05 was considered statistically significant.

We also analyzed the independent and combined effects of single-child status and ideal lifestyle category on components of CMRFs, including hyperglycemia, hypertension, dyslipidemia, and abdominal obesity as a supplementary result.

## Results

### Characteristics of participants

The characteristics of participants and their parents were showed in Table 1 and Supplementary Table 1. Overall, the prevalence of clustered CMRFs was 3.4%, with a higher prevalence in boys (4.0%) than girls (2.7%). Children and adolescents with clustered CMRFs had a higher proportion of single children (76.6 vs. 69.7%) and unfavorable lifestyles (62.1 vs. 29.2%) compared with their peers with non-clustered CMRFs. Children and adolescents with clustered CMRFs were more likely than those without clustered CMRFs to be male (61.1 vs. 50.8%), rural (50.2 vs. 41.1%), have a family history of diseases (18.9 vs. 12.5%), and have parents with lower proportion of high education level (24.9 vs. 30.3% for junior college or above).

**TABLE 1:** Demographic characteristics of eligible children and adolescents and their parents, stratified by clustered cardio-metabolic risk factors (CMRFs).

Characteristic	Total (*n* = 13,859)	Clustered CMRFs		*P*-value
		Yes (*n* = 470)	No (*n* = 13,389)	
Age*	12.2 (5.7)	12.2 (5.7)	12.7 (5.5)	<0.001
Boys	7,091 (51.2)	287 (61.1)	6,804 (50.8)	<0.001
Urban	8,117 (58.6)	234 (49.8)	7,883 (58.9)	<0.001
CMRFs				
High SBP (%)	1,090 (7.9)	247 (52.6)	843 (6.3)	<0.001
High DBP (%)	1,882 (13.6)	373 (79.4)	1,509 (11.3)	<0.001
Hypertension (%)	2,327 (16.8)	448 (95.3)	1,879 (14.0)	<0.001
High TC (%)	725 (5.2)	67 (14.3)	658 (4.9)	<0.001
High TG (%)	2,700 (19.5)	335 (71.3)	2,365 (17.7)	<0.001
High LDL-C (%)	379 (2.7)	47 (10.0)	332 (2.5)	<0.001
Low HDL-C (%)	1,343 (9.7)	201 (42.8)	1,142 (8.5)	<0.001
Dyslipidemia (%)	3,948 (28.5)	452 (96.2)	3,496 (26.1)	<0.001
High FBG (%)	258 (1.9)	55 (11.7)	203 (1.5)	<0.001
Abdominal obesity (%)	3,101 (22.4)	466 (99.1)	2,635 (19.7)	<0.001
Single children	9,688 (69.9)	360 (76.6)	9,328 (69.7)	0.001
Number of ideal lifestyle factors				<0.001
0–3 (unfavorable lifestyle)	4,199 (30.3)	292 (62.1)	3,907 (29.2)	
4 (intermediate lifestyle)	5,996 (43.3)	144 (30.6)	5,852 (43.7)	
5–7 (favorable lifestyle)	3,664 (26.4)	34 (7.2)	3,630 (27.1)	
Family history of diseases^$^	1,761 (12.7)	89 (18.9)	1,672 (12.5)	<0.001
Parental education level				0.036
Primary or below	385 (2.8)	12 (2.6)	373 (2.8)	
Secondary or equivalent	9,300 (67.1)	341 (72.5)	8,959 (66.9)	
Junior college or above	4,174 (30.1)	117 (24.9)	4,057 (30.3)	
Parental current tobacco consumption	7,991 (57.7)	273 (58.1)	7,718 (57.6)	0.849
Parental current alcohol consumption	3,935 (28.4)	129 (27.4)	3,806 (28.4)	0.643

*Quantitative variables are shown as median (interquartile range). ^$^Family history of diseases includes obesity, hypertension, diabetes mellitus and cerebrovascular disease. CMRFs, cardio-metabolic risk factors; SBP, systolic blood pressure; DBP, diastolic blood pressure; BP, blood pressure; TC, total cholesterol; TG, triglycerides; LDL-C, low density lipoprotein cholesterol; HDL-C, high density lipoprotein cholesterol; FBG, fasting blood glucose.

Children who were included in the final sample (*n* = 13,859) were more likely than those in the primary sample (*n* = 15,733) to be rural (41.4 vs. 38.2%). There were no statistically significant differences in age group and sex between the primary sample and the final sample (Supplementary Table 2).

### The independent effects of single-child status and lifestyle category on clustered cardio-metabolic risk factors

**FIGURE 2:**
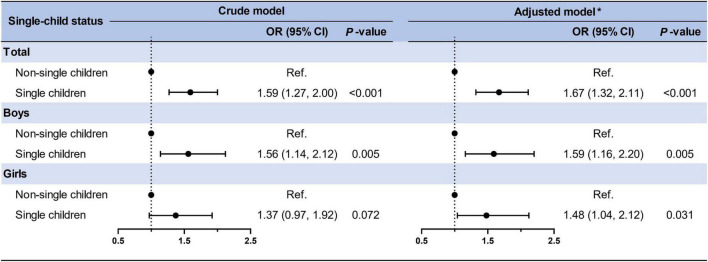
Association between single-child status and clustered CMRFs in children and adolescents. *Adjusted for age, residence, family history of diseases (obesity, hypertension, diabetes mellitus, and cerebrovascular disease), parental education level, parental tobacco, and alcohol consumption and the clustered effect of schools.

**FIGURE 3:**
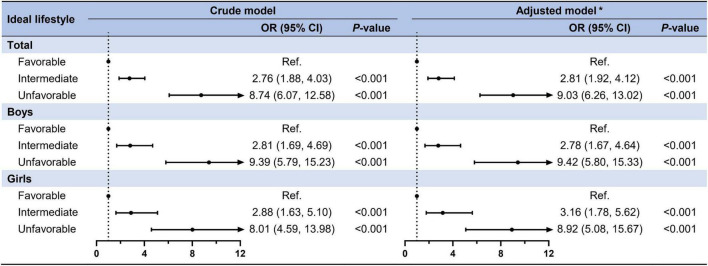
Association between ideal lifestyle factors and clustered CMRFs in children and adolescents. *Adjusted for age, residence, family history of diseases (obesity, hypertension, diabetes mellitus, and cerebrovascular disease), parental education level, parental tobacco, and alcohol consumption and the clustered effect of schools.

As presented in Figure 2, single children had a higher risk of CMRFs (adjusted model: OR = 1.67, 95% CI: 1.32–2.11) than non-single children in both crude and adjusted models. Although the sex interaction was not statistically significant, we stratified it by sex and found that this effect remained statistically significant for both boys (OR = 1.59, 95% CI: 1.16–2.20) and girls (OR = 1.48, 95% CI: 1.04–2.12). In the total population, participants engaged in intermediate (OR = 2.81, 95% CI: 1.92–4.12) and unfavorable lifestyles (OR = 9.03, 95% CI: 6.26–13.02) had a higher risk of clustered CMRFs compared to favorable lifestyles after adjusting for covariates (Figure 3). When stratified by sex, slightly higher OR values were observed in the unfavorable lifestyle group of boys (boys: OR = 9.42, 95% CI: 5.80–15.33 vs. girls: OR = 8.92, 95% CI: 5.08–15.67), although the difference was not statistically significant. The independent effects of single-child status and ideal lifestyle category on components of CMRFs have been shown in Supplementary Tables 3, 4.

### The combined effect of single-child status and lifestyle category on clustered cardio-metabolic risk factors

**FIGURE 4:**
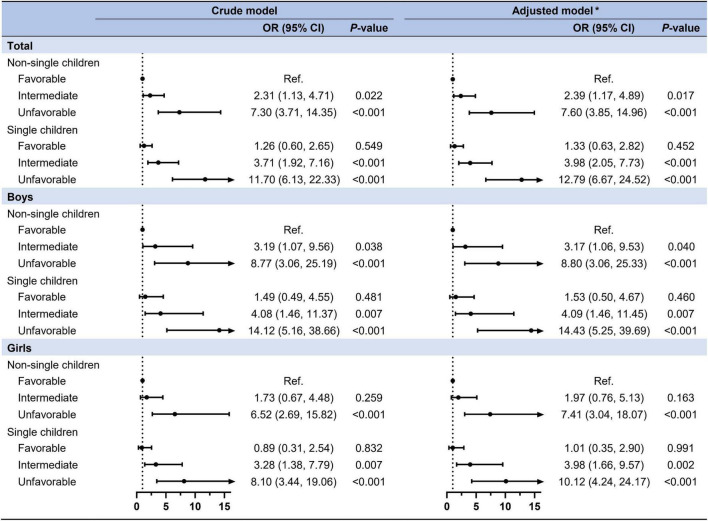
The combined effect of single-child status and ideal lifestyle on clustered CMRFs. *Adjusted for age, residence, family history of diseases (obesity, hypertension, diabetes mellitus, and cerebrovascular disease), parental education level, parental tobacco, and alcohol consumption and the clustered effect of schools.

As shown in Figure 4, a stronger combined effect of single children and ideal lifestyle category was found in clustered CMRFs. Compared to children and adolescents in the non-single children and favorable lifestyle group, an extremely high risk of clustered CMRFs was observed among those in the non-single children and unfavorable lifestyle group (OR = 7.60, 95% CI: 3.85–14.96) and single children and unfavorable lifestyle group (OR = 12.79, 95% CI: 6.67–24.52). No statistical significance was found in the single children and favorable lifestyle group (OR = 1.33, 95% CI: 0.63–2.82). When stratified by sex, these relationships remained consistent with the total population but with no statistical difference between boys and girls in the OR values of each subgroup. Supplementary Table 5 shows the combined effect of single-child status and ideal lifestyle category on components of CMRFs. In addition to high FBG, the combined effect of single children and ideal lifestyle category was statistically significant for hypertension, dyslipidemia and abdominal obesity, with the largest OR for abdominal obesity.

## Discussion

To the best of our knowledge, this study is the first to evaluate the combined effect of single children and lifestyle category on clustered CMRFs. According to the findings of this study, both single children and unfavorable lifestyles were associated with an increased risk of clustered CMRFs. The risk of clustered CMRFs increased significantly when single children and an unfavorable lifestyle were combined, which was almost neutralized when single children adhered to a favorable lifestyle. However, no sex difference was observed in this study.

The associations between the single children and an increased risk of elevated blood pressure (35), dyslipidemia (10), hypertension (13), abdominal obesity (11, 35), overweight and obesity (36, 37) were confirmed in China and European countries. However, one study found the opposite results that children with siblings were associated with a higher risk of hypertension (12), which the authors believed was likely attributed to adjustments for BMI or obesity status. The results of existing studies on lifestyles on CMRFs were more consistent, finding that an unfavorable lifestyle increased the risk of CMRFs or CVDs (38–40). Moreover, a meta-analysis showed that for each increase in ideal cardiovascular health as defined by the American Heart Association, cardiovascular mortality decreased by 19% (41). This present study added to the evidence that further emphasized the importance of adhering to a favorable lifestyle. No sex differences were found in this study, most likely due to the low prevalence of clustered CMRFs (3.4%) and, as a result, the relatively small number of children in each subgroup when stratified by sex, but indicated that intervention developed from single-child status or lifestyle perspectives or both would work for both sexes under such level of CMRFs.

Previous studies have identified an association between single-child families and components of CMRFs, however, the underlying mechanisms remain unknown. Even though the resource dilution theory (42, 43) contended that single children received more resources and care than children with siblings, the current nutritional and social environment may encourage the adoption of unhealthy lifestyles, which in turn resulted in metabolic abnormalities. Notably, this study discovered that when single children maintained a healthy lifestyle, the negative effect of single children on clustered CMRFs appeared to be offset, implying that the mechanism of health risks caused by single children was most likely due to their tendency to unfavorable lifestyles, such as fast food over intake (22), sugar excessive consumption (36), and physical inactivity (23). Based on the available evidence, the risks of being a single child might be mitigated by diluting the allocation of resources by expanding the family. On the other hand, children and adolescents should be encouraged to cultivate and maintain a healthy lifestyle, whether from single child or non-single child families.

To cope with the challenge of aging issue, the Chinese government ended one-child policy with the two-child policy in Zeng and Hesketh (44), and opened up the three-child policy in Jing et al. (45). However, the increasingly open policy has not improved China’s low fertility rate as expected (46, 47). According to the China Women’s Federation, the intention rate of parents to have a second child was 20.5% in 2017 (48), significantly lower than the 61.25% recorded under the one-child policy (49). Single children made up a large proportion of all structural families in an international environment of high income and low fertility (50). The level of delaying childbearing has been increasing, especially with the emergence of the second demographic transition (51), which suggested that this proportion was likely to increase in the future, and the resulting health problems should not be ignored. This study highlighted the need for the government and society to increase support for the birth of second and third children, such as reducing the financial burden and relieving the pressure of childrearing, and increasing couples’ free time. The findings of this study provided new evidence that single children with unfavorable lifestyles might be an important target group for future clustered CMRFs interventions, and it was necessary to strengthen the establishment of a friendly birth environment and health education at the family level to guide children toward a favorable lifestyle.

There were several limitations to the study. Firstly, this was a cross-sectional study with limited causal inference. Secondly, the lifestyle information of children and adolescents was entirely based on a questionnaire survey, which had a certain recall bias. Thirdly, although the method of imputation was used for missing variables, 12% of the samples were still excluded in this study, and thus there may be some selection bias in the final analyzed samples. However, when the background variables of the initial and final samples were compared, only urban and rural differences were discovered in this study. Fourthly, although some confounding factors were adjusted in this study, there was still residual confounding. Lastly, adding BMI as a scoring indicator in the definition of a healthy lifestyle may improve the correlation of lifestyle with clustered CMRFs, especially since abdominal obesity was included as a component of CMRFs in this study.

## Conclusion

Single children with unfavorable lifestyles were associated with an obvious risk of clustered CMRFs, which might be partially offset by expanding family size (the number of siblings) or establishing a favorable lifestyle. A birth-friendly social environment as well as a family environment with a favorable lifestyle are encouraged in China.

## Data availability statement

The data supporting the conclusions of this article can be made available from the corresponding author upon request.

## Ethics statement

The studies involving human participants were reviewed and approved by the Ethics Committee of Peking University (NO.IRB0000105213034). Written informed consent to participate in this study was provided by the participants’ legal guardian/next of kin.

## Author contributions

YD, ZZ, YM, YS, and JM conducted data collection and data management. JD and YS conducted manuscript design. JD, NM, and YL conducted statistical analysis. JD, NM, YL, PZ, DS, SC, YD, ZZ, and YM wrote and finalized the manuscript. YS and JM reviewed and revised the manuscript. All authors contributed to the article and approved the submitted version.
