# Supplementary material for: The independent and combined effects of single-child status and ideal lifestyle on clustered cardio-metabolic risk factors among Chinese children and adolescents
Source: Front Nutr. 2022 Aug 29;9:987334. doi: 10.3389/fnut.2022.987334 (PMC9464906; doi:10.3389/fnut.2022.987334)
Supplement: Supplementary file 1 [file Data_Sheet_1.docx]

Supplementary Material

**Table S1.** Demographic characteristics of eligible children and adolescents and their parents, stratified by sex.

| **Characteristic** | **Total**  **(n=13,859)** | **Sex** | | ***P*-value** |
| --- | --- | --- | --- | --- |
|  |  | **Boys**  **(n=7,091)** | **Girls**  **(n=6,768)** |  |
| Age ^*^ | 12.2 (5.7) | 12.1 (5.3) | 12.3 (5.9) | 0.072 |
| Urban | 8,117 (58.6) | 4,185 (59) | 3,932 (58.1) | 0.271 |
| CMRFs |  |  |  |  |
| High SBP, % | 1,090 (7.9) | 637 (9.0) | 453 (6.7) | <0.001 |
| High DBP, % | 1,882 (13.6) | 1,098 (15.5) | 784 (11.6) | <0.001 |
| Hypertension, % | 2,327 (16.8) | 1,365 (19.2) | 962 (14.2) | <0.001 |
| High TC, % | 725 (5.2) | 336 (4.7) | 389 (5.7) | 0.008 |
| High TG, % | 2,700 (19.5) | 1,244 (17.5) | 1,456 (21.5) | <0.001 |
| High LDL-C, % | 379 (2.7) | 177 (2.5) | 202 (3.0) | 0.078 |
| Low HDL-C, % | 1,343 (9.7) | 767 (10.8) | 576 (8.5) | <0.001 |
| Dyslipidemia, % | 3,948 (28.5) | 1,916 (27.0) | 2,032 (30.0) | <0.001 |
| High FBG, % | 258 (1.9) | 184 (2.6) | 74 (1.1) | <0.001 |
| Abdominal obesity, % | 3,101 (22.4) | 1,535 (21.6) | 1,566 (23.1) | 0.035 |
| Clustered CMRFs, % | 470 (3.4) | 287 (4.0) | 183 (2.7) | <0.001 |
| Single children | 9,688 (69.9) | 5,254 (74.1) | 4,434 (65.5) | <0.001 |
| Number of ideal lifestyle factors |  |  |  | <0.001 |
| 0–3 (unfavorable lifestyle) | 4,199 (30.3) | 2,363 (33.3) | 1,836 (27.1) |  |
| 4 (intermediate lifestyle) | 5,996 (43.3) | 2,834 (40.0) | 3,162 (46.7) |  |
| 5–7 (favorable lifestyle) | 3,664 (26.4) | 1,894 (26.7) | 1,770 (26.2) |  |
| Family history of diseases ^$^ | 1,761 (12.7) | 840 (11.8) | 921 (13.6) | 0.002 |
| Parental education level |  |  |  | <0.001 |
| Primary or below | 385 (2.8) | 190 (2.7) | 195 (2.9) |  |
| Secondary or equivalent | 9,300 (67.1) | 4,871 (68.7) | 4,429 (65.4) |  |
| Junior college or above | 4,174 (30.1) | 2,030 (28.6) | 2,144 (31.7) |  |
| Parental current tobacco consumption | 7,991 (57.7) | 4,171 (58.8) | 3,820 (56.4) | 0.005 |
| Parental current alcohol consumption | 3,935 (28.4) | 1,975 (27.9) | 1,960 (29.0) | 0.148 |

^*^ Quantitative variables are shown as median (interquartile range). ^$^ Family history of diseases includes obesity, hypertension, diabetes mellitus and cerebrovascular disease. CMRFs, cardio-metabolic risk factors; SBP, systolic blood pressure; DBP, diastolic blood pressure; BP, blood pressure; TC, total cholesterol; TG, triglycerides; LDL-C, low density lipoprotein cholesterol; HDL-C, high density lipoprotein cholesterol; FBG, fasting blood glucose.

| **Characteristic** | **Primary sample**  **(n=15,733)** | **Final sample**  **(n=13,859)** | **χ^2^** | ***P*-value** |
| --- | --- | --- | --- | --- |
| Age group |  |  | 1.385 | 0.239 |
| 7–12 years | 9589 (60.9) | 8354 (60.3) |  |  |
| 13–18 years | 6144 (39.1) | 5505 (39.7) |  |  |
| Sex |  |  | 0.183 | 0.669 |
| Boys | 8089 (51.4) | 7091 (51.2) |  |  |
| Girls | 7644 (48.6) | 6768 (48.8) |  |  |
| Residence |  |  | 32.780 | <0.001 |
| Urban | 9728 (61.8) | 8117 (58.6) |  |  |
| Rural | 6005 (38.2) | 5742 (41.4) |  |  |

**Table S2.** The comparison between the primary sample and the final sample.

**Table S3.** Association between single-child status and CMRFs components in children and adolescents.

| **Single-child status** | | **Crude model** | |  | **Adjusted model ^*^** | |
| --- | --- | --- | --- | --- | --- | --- |
|  |  | **OR (95% CI)** | ***P*-value** |  | **OR (95% CI)** | ***P*-value** |
| High FBG | |  |  |  |  |  |
|  | Non-single children | Ref. |  |  | Ref. |  |
|  | Single children | 1.10 (0.82, 1.48) | 0.542 |  | 1.08 (0.79, 1.47) | 0.620 |
| High SBP | |  |  |  |  |  |
|  | Non-single children | Ref. |  |  | Ref. |  |
|  | Single children | **1.18 (1.02, 1.36)** | 0.026 |  | **1.19 (1.03, 1.39)** | 0.021 |
| High DBP | |  |  |  |  |  |
|  | Non-single children | Ref. |  |  | Ref. |  |
|  | Single children | **1.20 (1.07, 1.35)** | 0.002 |  | **1.19 (1.05, 1.35)** | 0.005 |
| High BP | |  |  |  |  |  |
|  | Non-single children | Ref. |  |  | Ref. |  |
|  | Single children | **1.24 (1.11, 1.38)** | <0.001 |  | **1.23 (1.10, 1.38)** | <0.001 |
| High TC | |  |  |  |  |  |
|  | Non-single children | Ref. |  |  | Ref. |  |
|  | Single children | 1.17 (0.98, 1.41) | 0.086 |  | 1.15 (0.95, 1.39) | 0.146 |
| High TG | |  |  |  |  |  |
|  | Non-single children | Ref. |  |  | Ref. |  |
|  | Single children | 1.05 (0.93, 1.18) | 0.436 |  | 1.05 (0.93, 1.18) | 0.460 |
| High LDL-C | |  |  |  |  |  |
|  | Non-single children | Ref. |  |  | Ref. |  |
|  | Single children | 1.29 (1.00, 1.66) | 0.050 |  | 1.23 (0.95, 1.60) | 0.119 |
| Low HDL-C | |  |  |  |  |  |
|  | Non-single children | Ref. |  |  | Ref. |  |
|  | Single children | **1.22 (1.06, 1.40)** | 0.005 |  | **1.25 (1.08, 1.44)** | 0.002 |
| Dyslipidemia | |  |  |  |  |  |
|  | Non-single children | Ref. |  |  | Ref. |  |
|  | Single children | 1.09 (0.99, 1.20) | 0.090 |  | 1.10 (1.00, 1.22) | 0.058 |
| Abdominal obesity | |  |  |  |  |  |
|  | Non-single children | Ref. |  |  | Ref. |  |
|  | Single children | **1.26 (1.14, 1.38)** | <0.001 |  | **1.25 (1.13, 1.38)** | <0.001 |

**^*^** Adjusted for age, sex, residence, family history of diseases (obesity, hypertension, diabetes mellitus and cerebrovascular disease), parental education level, parental tobacco and alcohol consumption.

**Table S4.** Association between Ideal lifestyle category and CMRFs components in children and adolescents.

| **Ideal lifestyle group** | | **Crude model** | |  | **Adjusted model ^*^** | |
| --- | --- | --- | --- | --- | --- | --- |
|  |  | **OR (95% CI)** | ***P*-value** |  | **OR (95% CI)** | ***P*-value** |
| High FBG | |  |  |  |  |  |
|  | Favorable | Ref. |  |  | Ref. |  |
|  | Intermediate | 1.00 (0.73, 1.37) | 0.996 |  | 1.00 (0.72, 1.37) | 0.981 |
|  | Unfavorable | 1.11 (0.79, 1.57) | 0.542 |  | 1.12 (0.79, 1.58) | 0.527 |
| High SBP | |  |  |  |  |  |
|  | Favorable | Ref. |  |  | Ref. |  |
|  | Intermediate | **1.34 (1.12, 1.60)** | 0.001 |  | **1.39 (1.16, 1.65)** | <0.001 |
|  | Unfavorable | **2.27 (1.91, 2.71)** | <0.001 |  | **2.37 (1.98, 2.83)** | <0.001 |
| High DBP | |  |  |  |  |  |
|  | Favorable | Ref. |  |  | Ref. |  |
|  | Intermediate | **1.21 (1.05, 1.39)** | 0.008 |  | **1.21 (1.05, 1.39)** | 0.008 |
|  | Unfavorable | **1.78 (1.54, 2.05)** | <0.001 |  | **1.77 (1.54, 2.05)** | <0.001 |
| High BP | |  |  |  |  |  |
|  | Favorable | Ref. |  |  | Ref. |  |
|  | Intermediate | **1.24 (1.09, 1.40)** | 0.001 |  | **1.25 (1.10, 1.42)** | 0.001 |
|  | Unfavorable | **1.88 (1.65, 2.15)** | <0.001 |  | **1.91 (1.67, 2.18)** | <0.001 |
| High TC | |  |  |  |  |  |
|  | Favorable | Ref. |  |  | Ref. |  |
|  | Intermediate | **1.29 (1.07, 1.57)** | 0.008 |  | **1.34 (1.10, 1.62)** | 0.003 |
|  | Unfavorable | **1.24 (1.01, 1.53)** | 0.043 |  | **1.31 (1.06, 1.62)** | 0.013 |
| High TG | |  |  |  |  |  |
|  | Favorable | Ref. |  |  | Ref. |  |
|  | Intermediate | **1.36 (1.18, 1.56)** | <0.001 |  | **1.37 (1.19, 1.58)** | <0.001 |
|  | Unfavorable | **2.14 (1.86, 2.46)** | <0.001 |  | **2.15 (1.86, 2.48)** | <0.001 |
| High LDL-C | |  |  |  |  |  |
|  | Favorable | Ref. |  |  | Ref. |  |
|  | Intermediate | **1.55 (1.18, 2.03)** | 0.002 |  | **1.60 (1.22, 2.11)** | 0.001 |
|  | Unfavorable | **1.60 (1.19, 2.14)** | 0.002 |  | **1.69 (1.25, 2.26)** | 0.001 |
| Low HDL-C | |  |  |  |  |  |
|  | Favorable | Ref. |  |  | Ref. |  |
|  | Intermediate | **1.31 (1.11, 1.53)** | 0.001 |  | **1.28 (1.09, 1.50)** | 0.003 |
|  | Unfavorable | **1.69 (1.43, 2.00)** | <0.001 |  | **1.64 (1.39, 1.94)** | <0.001 |
| Dyslipidemia | |  |  |  |  |  |
|  | Favorable | Ref. |  |  | Ref. |  |
|  | Intermediate | **1.32 (1.19, 1.47)** | <0.001 |  | **1.32 (1.18, 1.47)** | <0.001 |
|  | Unfavorable | **1.77 (1.58, 1.99)** | <0.001 |  | **1.77 (1.57, 1.98)** | <0.001 |
| Abdominal obesity | |  |  |  |  |  |
|  | Favorable | Ref. |  |  | Ref. |  |
|  | Intermediate | **2.32 (2.03, 2.66)** | <0.001 |  | **2.40 (2.09, 2.75)** | <0.001 |
|  | Unfavorable | **8.86 (7.74, 10.14)** | <0.001 |  | **9.35 (8.15, 10.72)** | <0.001 |

**^*^** Adjusted for age, sex, residence, family history of diseases (obesity, hypertension, diabetes mellitus and cerebrovascular disease), parental education level, parental tobacco and alcohol consumption. FBG, fasting blood glucose; SBP, systolic blood pressure; DBP, diastolic blood pressure; BP, blood pressure; TC, total cholesterol; TG, triglycerides; LDL-C, low density lipoprotein cholesterol; HDL-C, high density lipoprotein cholesterol; CMRFs, cardio-metabolic risk factors.

**Table S5.** The combined effect of single-child status and ideal lifestyle category on CMRFs components.

| **Subgroup** | |  | **OR (95% CI) ^*^** | ***P*-value** |
| --- | --- | --- | --- | --- |
| **High FBG** | |  |  |  |
| Non-single children | Favorable |  | Ref. |  |
|  | Intermediate |  | 0.87 (0.50, 1.52) | 0.621 |
|  | Unfavorable |  | 0.94 (0.50, 1.75) | 0.835 |
| Single children | Favorable |  | 0.92 (0.54, 1.57) | 0.759 |
|  | Intermediate |  | 0.98 (0.60, 1.61) | 0.940 |
|  | Unfavorable |  | 1.12 (0.67, 1.87) | 0.677 |
| **High SBP** | |  |  |  |
| Non-single children | Favorable |  | Ref. |  |
|  | Intermediate |  | 1.42 (1.05, 1.94) | 0.025 |
|  | Unfavorable |  | **2.31 (1.69, 3.16)** | <0.001 |
| Single children | Favorable |  | 1.19 (0.87, 1.61) | 0.272 |
|  | Intermediate |  | **1.62 (1.22, 2.15)** | 0.001 |
|  | Unfavorable |  | **2.83 (2.13, 3.76)** | <0.001 |
| **High DBP** | |  |  |  |
| Non-single children | Favorable |  | Ref. |  |
|  | Intermediate |  | 1.04 (0.82, 1.33) | 0.740 |
|  | Unfavorable |  | **1.57 (1.22, 2.01)** | <0.001 |
| Single children | Favorable |  | 1.01 (0.80, 1.29) | 0.917 |
|  | Intermediate |  | **1.32 (1.05, 1.64)** | 0.015 |
|  | Unfavorable |  | **1.91 (1.52, 2.39)** | <0.001 |
| **High BP** | |  |  |  |
| Non-single children | Favorable |  | Ref. |  |
|  | Intermediate |  | 1.10 (0.88, 1.38) | 0.400 |
|  | Unfavorable |  | **1.64 (1.30, 2.07)** | <0.001 |
| Single children | Favorable |  | 1.05 (0.84, 1.31) | 0.651 |
|  | Intermediate |  | **1.39 (1.14, 1.70)** | 0.001 |
|  | Unfavorable |  | **2.14 (1.74, 2.63)** | <0.001 |
| **High TC** | |  |  |  |
| Non-single children | Favorable |  | Ref. |  |
|  | Intermediate |  | **1.53 (1.03, 2.26)** | 0.035 |
|  | Unfavorable |  | **1.64 (1.08, 2.50)** | 0.021 |
| Single children | Favorable |  | **1.36 (0.94, 1.97)** | 0.102 |
|  | Intermediate |  | **1.74 (1.22, 2.47)** | 0.002 |
|  | Unfavorable |  | **1.65 (1.14, 2.38)** | 0.008 |
| **High TG** | |  |  |  |
| Non-single children | Favorable |  | Ref. |  |
|  | Intermediate |  | **1.47 (1.14, 1.88)** | 0.003 |
|  | Unfavorable |  | **2.21 (1.71, 2.86)** | <0.001 |
| Single children | Favorable |  | 1.10 (0.86, 1.40) | 0.442 |
|  | Intermediate |  | **1.46 (1.17, 1.84)** | 0.001 |
|  | Unfavorable |  | **2.33 (1.85, 2.93)** | <0.001 |
| **High LDL-C** | |  |  |  |
| Non-single children | Favorable |  | Ref. |  |
|  | Intermediate |  | **3.06 (1.56, 5.99)** | 0.001 |
|  | Unfavorable |  | **3.29 (1.63, 6.62)** | 0.001 |
| Single children | Favorable |  | **2.38 (1.24, 4.55)** | 0.009 |
|  | Intermediate |  | **3.26 (1.74, 6.12)** | <0.001 |
|  | Unfavorable |  | **3.39 (1.79, 6.44)** | <0.001 |
| **Low HDL-C** | |  |  |  |
| Non-single children | Favorable |  | Ref. |  |
|  | Intermediate |  | 1.34 (0.99, 1.80) | 0.056 |
|  | Unfavorable |  | **1.66 (1.22, 2.27)** | 0.001 |
| Single children | Favorable |  | 1.29 (0.96, 1.73) | 0.091 |
|  | Intermediate |  | **1.61 (1.23, 2.12)** | 0.001 |
|  | Unfavorable |  | **2.09 (1.59, 2.77)** | <0.001 |
| **Dyslipidemia** | |  |  |  |
| Non-single children | Favorable |  | Ref. |  |
|  | Intermediate |  | **1.43 (1.18, 1.75)** | <0.001 |
|  | Unfavorable |  | **1.83 (1.48, 2.26)** | <0.001 |
| Single children | Favorable |  | 1.17 (0.97, 1.42) | 0.104 |
|  | Intermediate |  | **1.49 (1.25, 1.79)** | <0.001 |
|  | Unfavorable |  | **2.03 (1.69, 2.45)** | <0.001 |
| **Abdominal obesity** | |  |  |  |
| Non-single children | Favorable |  | Ref. |  |
|  | Intermediate |  | **2.28 (1.76, 2.96)** | <0.001 |
|  | Unfavorable |  | **8.81 (6.83, 11.37)** | <0.001 |
| Single children | Favorable |  | 1.17 (0.90, 1.52) | 0.245 |
|  | Intermediate |  | **2.86 (2.25, 3.64)** | <0.001 |
|  | Unfavorable |  | **11.18 (8.80, 14.21)** | <0.001 |

**^*^** Adjusted for age, sex, residence, family history of diseases (obesity, hypertension, diabetes mellitus and cerebrovascular disease), parental education level, parental tobacco and alcohol consumption. CMRFs, cardio-metabolic risk factors; FBG, fasting blood glucose; SBP, systolic blood pressure; DBP, diastolic blood pressure; BP, blood pressure; TC, total cholesterol; TG, triglycerides; LDL-C, low density lipoprotein cholesterol; HDL-C, high density lipoprotein cholesterol.
